# Optimal Dietary α-Starch Requirement and Its Effects on Growth and Metabolic Regulation in Chinese Hook Snout Carp (*Opsariichthys bidens*)

**DOI:** 10.3390/biology14121687

**Published:** 2025-11-26

**Authors:** Wenjing Cai, Xiaonian Luo, Jiao Li, Youjian Duan, Yong Wei, Yuxin Xing, Zongyun Hu, Chunyue Zhu

**Affiliations:** 1 College of Fisheries and Life, Dalian Ocean University, Dalian 116023, China; wenjng_cai@outlook.com (W.C.); luoxiaonian@dlou.edu.cn (X.L.); youjianduan@dlou.edu.cn (Y.D.); weiyong0608@gmail.com (Y.W.); xingyuxin1997@163.com (Y.X.); 2 Key Laboratory of Mariculture & Stock Enhancement in North China’s Sea, Ministry of Agriculture, Dalian Ocean University, Dalian 116023, China; 3 Liaoning Key Laboratory of Aquatic Animal Diseases Control, Liaoning Institute of Freshwater Fisheries, Liaoyang 111000, China; huzongyun2004@163.com (Z.H.); 18747551907@163.com (C.Z.)

**Keywords:** dietary carbohydrate, *Opsariichthys bidens*, dietary requirement, glucose metabolism, lipid metabolism

## Abstract

This study investigated the effects of dietary carbohydrate levels on growth performance, body composition, and hepatic expression of metabolic genes in Chinese hook snout carp (*Opsariichthys bidens*). Fish were fed five isonitrogenous diets with graded α-starch levels (8%, 14%, 20%, 26%, and 32%) for 56 days. The diet containing 14% α-starch significantly increased the weight gain rate (WGR) and specific growth rate (SGR) of *O. bidens* (*p* < 0.05). Both broken-line and polynomial regression analyses on WGR and SGR consistently indicated an optimal dietary α-starch level of approximately 14–17%. High carbohydrate diets significantly elevated plasma glucose, triglyceride, and cholesterol levels, as well as hepatosomatic and intraperitoneal fat indices. Gene expression analysis revealed that moderate carbohydrate intake upregulated lipoprotein lipase (*lpl*), hormone-sensitive lipase (*hsl)*, and carnitine palmitoyltransferase 1 (*cpt1*) gene expressions, enhancing lipolysis and β-oxidation, whereas excessive carbohydrate intake (>26% α-starch) suppressed these pathways but strongly induced *acc1* gene expressions, promoting lipogenesis. Additionally, glycogen metabolism genes (glycogen synthase (*gys*) and glycogen phosphorylase (*pyg*) and glycolysis-related phosphofructokinase (*pfk*) were responsive to carbohydrate supply, while oxidative metabolism gene *cs* was downregulated under excessive carbohydrate, implying reduced mitochondrial oxidative metabolism. Overall, *O. bidens* exhibited limited carbohydrate utilization, with optimal intake supporting growth and metabolic balance, whereas excessive intake redirected glucose toward glycogen and lipid accumulation, leading to metabolic imbalance.

## Simple Summary

Incorporating suitable levels of carbohydrates into fish feed can help reduce feed costs and spare protein for growth. However, the optimal carbohydrate requirement for Chinese hook snout carp (*Opsariichthys bidens*) has not been clarified. Both broken-line and polynomial regression analyses on WGR and SGR consistently indicated an optimal dietary α-starch level of approximately 14–17%, confirming that moderate carbohydrate intake supports optimal growth and enhanced lipolysis and β-oxidation in *Opsariichthys bidens*. In contrast, excessive carbohydrate intake (>26% α-starch) suppresses these pathways and stimulates lipogenesis, leading to elevated plasma glucose and lipids, as well as hepatic glycogen and lipid accumulation. These findings indicate that *O. bidens* can utilize only a limited amount of carbohydrates efficiently. Properly balancing dietary carbohydrates is therefore crucial for maintaining metabolic health and improving aquaculture productivity in this species.

## 1. Introduction

The Chinese hook snout carp (*Opsariichthys bidens*) is a small endemic Cypriniformes minnow in East Asia. It is widely distributed in most major river systems in China, as well as in the rivers of North Korea and Japan [1]. This species is typically found in relatively small streams or tributaries of large rivers, particularly fast-flowing montane streams with gravel substrates and low water temperatures. In recent years, *O. bidens* has been farmed in several regions of China, such as the Zhejiang and Liaoning Provinces. Owing to its rapid growth, high nutritional value, desirable taste, and significant economic potential, *O. bidens* has transformed from being regarded as an economically harmful species to an emerging aquaculture fish species. To ensure stable production and high-quality supply of *O. bidens* for the market, it is essential to better understand the factors influencing its reproductive biology, growth rate, nutritional composition, taste, and muscle texture.

Preliminary diet analyses have suggested that *O. bidens* exhibits ontogenetic dietary shifts [2,3]. Individuals smaller than 140 mm in body length primarily feed on small invertebrates, intermediate-sized individuals feed mainly on large invertebrates, and large individuals fed on other small fish and fish larvae. Because of the presence of insects and fish remains in the intestines of wild *O. bidens,* it is regarded as a small carnivorous fish. To date, the role of external environmental factors, especially the nutritional requirements of *O. bidens*, has received little attention, even though it plays a critical role in influencing the growth, development, and nutritional quality of *O. bidens*. A previous study estimated the dietary protein requirement of *O. bidens* to be approximately 42.97% [4]. Variations in dietary protein levels significantly affect the contents of certain fatty acids in its muscle [4]. However, little is known about appropriate carbohydrate levels or their effects on growth and metabolism in this species.

Carbohydrates are the most abundant and economically indispensable source of nutrients in fish feed formulation [5]. Optimal carbohydrate levels in fish diets can be metabolized into amino acids, lipids, and nucleotides to maintain nutritional metabolism, thereby sparing protein and lipid consumption in the diets; thus, it can lead to lower cost of diet [6]. α-starch is a product of cereal processing and has been widely used in aquafeeds as one of the main carbohydrates. Its amylopectin structure is more easily broken down by digestive enzymes, providing quick energy for aquatic species. Notably, α-starch has been proven to act as a natural binder and facilitate extruded pellet expansion [7]. From this perspective, dietary α-starch content should be maintained at a certain level for the manufacture of floating expanded feed.

However, compared to mammals, fish are generally considered to have a poor ability to utilize and a low tolerance for dietary carbohydrates [5,8]. Excessive dietary carbohydrates which cannot be efficiently utilized by fish negatively affect fish health. They can cause metabolic disorders and lead to physiological alterations, including prolonged hyperglycemia [9], hepatic anti-oxidative response [10], and high fat deposition in the liver [11]. It has been demonstrated that the utilization of dietary carbohydrates of teleost is species-specific, and in general, the glucose utilization capacity of carnivorous fish is relatively limited compared with omnivorous and herbivorous fish [8]. As *O. bidens* is a small carnivorous fish with limited capacity to utilize carbohydrates compared with herbivorous and omnivorous fishes [12,13], understanding its carbohydrate tolerance is crucial for feed formulation. To date, no systematic studies have evaluated suitable carbohydrate levels or their metabolic effects in this species. This study is designed to determine the optimal content of α-starch as a carbohydrate source in *O. bidens* diets. Furthermore, it investigates the effects of different α-starch levels on carbohydrate and lipid metabolism, providing a basis for developing nutritionally balanced and cost-effective feeds for sustainable aquaculture of *O. bidens*.

## 2. Materials and Methods

### 2.1. Ethical Approval

This experimental trial was approved by the Animal Ethics Committee of Dalian Ocean University (Permit Number: DLOU2023008) and performed in accordance with the relevant institutional and national guidelines, and the manuscript conformed to the ARRIVE Guidelines for Reporting Animal Research.

### 2.2. Experimental Diets

Five experimental diets containing different levels of α-starch (8%, 14%, 20%, 26%, and 32%) were formulated in the laboratory and named as C1, C2, C3, C4, and C5, respectively. The design of the feed formulas followed the guidelines described in the NRC (2011) [6] and previous studies on fish nutritional requirements [14,15,16]. The dietary protein content was formulated according to the protein requirement reported [4]. The formulation and analyzed composition of the diets are presented in Table 1. All ingredients were sourced from Coland Co., Ltd. (Wuhan, China). After thorough mixing, the diets were pelleted using a laboratory pelletizer (1.5 mm diameter) at an outlet temperature of 72 ± 2 °C, within 30 min after mixing. To ensure consistency and minimize oxidation, all diet ingredients, including fish oil and soybean oil, were obtained from the same batch from a commercial supplier and were stored at −20 °C prior to feed preparation. Following pelleting and air-drying, all experimental diets were also stored at −20 °C and used within 8 weeks. Under these controlled conditions, the risk of lipid oxidation was minimal; therefore, no additional antioxidant additives were included in the formulations. The use of casein, fish meal, α-starch, and cellulose, which are standard ingredients in fish nutrition research, allowed us to maintain stable nutrient profiles and to isolate the effect of dietary α-starch levels without confounding variations from other dietary components.

### 2.3. Fish and Feeding Trial

Chinese hook snout carp were obtained from Xingda Aquafarm (Liaoyang, China). Before the feeding trial, 500 fish were temporarily cultured in 10 tanks (1000 L) for a 7-day acclimation period, during which they were gradually transitioned to artificial diets. After the acclimation, the initial body weight and total length of all fish were recorded. The fish were then randomly allotted into five experimental groups, with each group containing three randomly assigned replicate tanks. In total, 15 tanks were used in the experiment, with 30 fish (initial weight: 2.80 ± 0.07 g) stocked in each tank. During the experimental period, filtered flow-through pond water was supplied to the tanks at a constant rate of 3 L/min and maintained at the following parameters: water temperature remained stable within a range of 25 °C to 27 °C, dissolved oxygen was maintained between 8.25 and 8.76 mg L^−1^, ammonia concentration remained within 0.10 ± 0.02 mg/L, and pH values ranged from 7.20 to 7.60. The fish were fed to apparent satiation twice daily at 08:00 and 17:00 throughout the 56-day feeding trial. For each tank, uneaten feed was collected after each feeding, dried, and subsequently used to calculate the feed intake.

### 2.4. Sampling

At the end of the feeding trial, the fish were fasted for 24 h before being anesthetized with MS-222 (Sigma-Aldrich, St. Louis, MO, USA; 50 mg/L in water). Final body weight and length of all fish were recorded prior to dissection. Six fish were randomly selected from each treatment, and blood samples were drawn from the caudal vein and allowed to stand at room temperature for 2 h. Serum was then separated by centrifugation at 3500× *g* for 10 min and stored at −80 °C until further use. The mass of the viscera, liver, mesenteric fat and intestine were carefully separated and weighed. Liver samples were immediately frozen in liquid nitrogen and stored at −80 °C for subsequent analysis. Dorsal muscle samples were collected after removing the scales and skin.

### 2.5. Sample Analysis

The proximate compositions (moisture, crude protein, crude lipid, and ash) of the experimental diets, as well as the whole body and muscle of fish, were determined following AOAC methods. Moisture was determined by oven drying at 105 °C until a constant weight was obtained. Crude protein content (N × 6.25) was determined according to the Kjeldahl method after acid digestion using an Auto Kjeldahl System (1030-Auto-analyzer; Tecator, Höganäs, Sweden). Crude lipid was determined by the ether extraction method using a Soxtec extraction System HT (Soxtec System HT6, Tecator, Höganäs, Sweden). Ash content was determined after samples were placed in a muffle furnace (OTF-1200X; Kejing, Hefei, China) at 550 °C for 4 h. Gross energy was obtained by means of an adiabatic bomb calorimeter (model WHR-15; Changsha, China, calibrated with benzoic acid).

Plasma glucose, cholesterol, and triglyceride contents were measured using the glucose oxidase method, the enzymatic (cholesterol oxidase) and colorimetric method, and the enzymatic (glycerol phosphate oxidase) and colorimetric (PAP) method, respectively, using test kits purchased from Nanjing Jiancheng, Nanjing, China. High-density lipoprotein (HDL) and low-density lipoprotein (LDL) were assayed using spectrophotometric procedures with the commercial kit (Nanjing Jiancheng, China) method.

### 2.6. RNA Extraction and cDNA Synthesis

After 8 weeks of feeding, six fish from each group were dissected and small pieces of liver samples were immediately frozen in liquid nitrogen for RNA extraction. Total RNA was extracted using the RNAiso Plus reagent (Takara, Kusatsu, Japan) according to the manufacturer’s instructions. The quality, quantity, and integrity of the RNA were determined by BioTek Synergy™ 2 (BioTek, Winooski, VT, USA) and agarose gel electrophoresis methods. For cDNA synthesis, 1 μg of total RNA was reverse-transcribed using SuperScript™ II Reverse Transcriptase (Takara, Kusatsu, Japan) in a total reaction volume of 20 μL, following the manufacturer’s guidelines.

### 2.7. Real-Time PCR Assays (qRT-PCR)

Information on primers used in this experiment are shown in Table 2. The primers were based on the RNA-Seq data which were in the NCBI SRA database (accession number PRJNA902523). The specificity of the primers was determined through sequencing and the melting curve of PCR products. Real-time PCR assays (qRT-PCR) were conducted on a MyiQ™ 2 Two-Color Real-Time PCR Detection System (BIO-RAD, Hercules, CA, USA) with AceQ^®^ qPCR SYBR^®^ Green Master Mix (Vazyme Biotech, Nanjing, China). The PCR cycling parameters were 95 °C for 3 mins, then followed by 40 cycles at 95 °C for 10 s, and 30 s at annealing temperature. After amplification, melting curve assay was performed. The temperature gradually increased from 65 °C to 95 °C, with 0.5 °C every 6 s. The mRNA expression levels were quantified relative to the expression of *β-actin* as the endogenous reference and analyzed by the optimized comparative Ct (2^−ΔΔCt^) value method [17].

### 2.8. Statistical Analysis

The following parameters were calculated to assess growth, feed efficiency, and fitness: weight gain rate (WGR), specific growth rate (SGR), food intake (FI), feed conversion ratio (FCR), viscerosomatic index (VSI), hepatosomatic index (HSI), intestosomatic index (ISI), and intraperitoneal fat ratio (IPF). These were calculated using the formulas provided below:

WGR = 100 × (final weight − initial weight)/initial weight;
SGR = 100 × (ln (final weight) − ln (initial weight))/days;
FI = Total food intake/(initial fish number × survival rate × culture days)
FCR = 100 × total food intake/weight gain;
VSI = 100 × (viscera weight/whole body weight);
HSI = 100 × (liver weight/whole body weight);
ISI = 100 × (gut weight/whole body weight);
IPF = 100 × (intraperitoneal fat weight/body weight).

All statistical analysis was performed with IBM SPSS Statistics (version 19.0). The normal distribution of variables and homoscedasticity were analyzed by Kolmogorov–Smirnov and Levene tests. One-way analysis of variance was conducted to compare the groups. Broken-line and polynomial regression models were performed to estimate the optimal dietary α-starch level based on WGR and SGR. Statistical significance was considered at *p* < 0.05. When significant differences were observed, Duncan’s test was conducted for multiple comparisons. All data were presented as mean ± SE (standard error).

## 3. Results

### 3.1. Growth Performance and Food Intake

**Figure 1:**
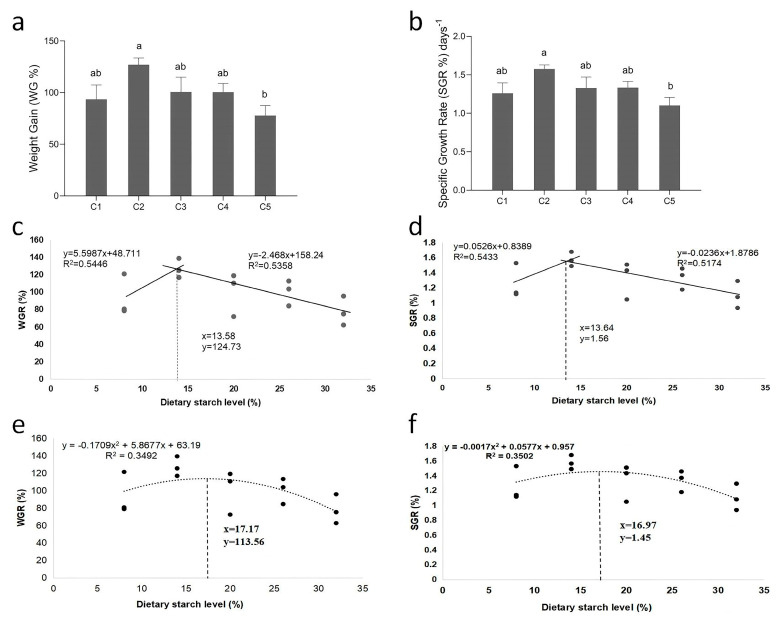
The growth performance of *O. bidens* fed different diets. (**a**) The weight gain rate of *O. bidens*. (**b**) The specific growth rate of *O. bidens*. Broken-line regression analysis showing the relationships between dietary α-starch level (%) and (**c**) weight gain rate (WGR, %) and (**d**) specific growth rate (SGR, %) of *O. bidens*. Polynomial regression analysis showing the relationships between dietary α-starch level (%) and (**e**) weight gain rate (WGR, %) and (**f**) specific growth rate (SGR, %) of *O. bidens*. Fish fed diets containing different levels of α-starch (8%, 14%, 20%, 26%, and 32%) were named as group C1, C2, C3, C4, and C5, respectively. Data are presented as mean ± SE of three replicates (*n* = 30). The bar with different letters indicates significant differences between groups based on one-way analysis of variance (ANOVA) followed by Duncan’s multiple range test (*p* < 0.05).

The growth performance of *O. bidens* fed diets with different carbohydrate levels is presented in Figure 1. Analysis of weight gain (WGR) and specific growth rate (SGR) revealed significant differences among the experimental groups. The results of WGR and SGR indicated an initial increase followed by a decrease, with the C2 group (14% α-starch) exhibiting the highest value of WGR and SGR, and C5 (32% α-starch) showing the lowest values (*p* < 0.05) (Figure 1a,b). To further validate the estimation of optimal dietary α-starch levels, both broken-line and polynomial regression analyses were performed using WGR and SGR as response variables. The broken-line model based on WGR and SGR indicated an optimal α-starch level of approximately 13.58–13.64% (Figure 1c,d), whereas the polynomial regression analysis based on WGR and SGR yielded a comparable estimate of 17.17 and 16.97% (Figure 1d,f). Despite the slight numerical difference, both analytical approaches revealed a similar growth performance of *O. bidens* that increased with dietary α-starch level up to an optimal range (approximately 14–17%), beyond which excessive starch inclusion led to a decline in growth rate.

**Figure 2:**
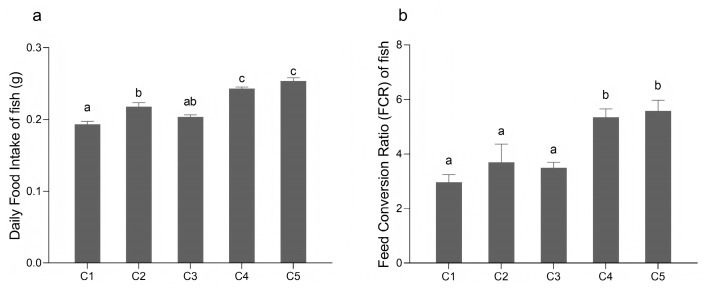
The food intake of *O. bidens* fed different diets. (**a**) The daily food intake of *O. bidens*. (**b**) The feed conversion ratio of *O. bidens*. Fish fed diets containing different levels of α-starch (8%, 14%, 20%, 26%, and 32%) were named as group C1, C2, C3, C4, and C5, respectively. Data are presented as mean ± SE of three tanks per group (*n* = 3). The bar with different letters indicates significant differences between groups based on one-way analysis of variance (ANOVA) followed by the Duncan’s multiple range test (*p* < 0.05).

Conversely, the food intake of *O. bidens* exhibited an increase as dietary carbohydrate levels increased. The C4 (26% α-starch) and C5 groups exhibited significantly higher food intake than the C1 (8% α-starch), C2, and C3 (20% α-starch) groups (*p* < 0.05) (Figure 2a). Similarly, FCR values followed the same pattern, with the C4 and C5 groups displaying significantly higher FCR than the C1, C2, and C3 groups (*p* < 0.05) (Figure 2b).

### 3.2. Whole Body and Tissue Composition

The body and muscle composition of *O. bidens* fed diets containing different carbohydrate levels are presented in Table 3. In terms of body composition, there were no significant differences observed in the moisture and ash content among the groups. The crude protein content in the C5 group was significantly higher compared to that of other groups (*p* < 0.05). An increase in crude protein content was observed from the C1 to C3 groups as dietary carbohydrate levels increased. However, the C4 group exhibited the lowest crude protein content. Crude lipid content was also highest in the C5 group, followed by the C2, C3, and C4 groups, with the C1 group showing the lowest value (*p* < 0.05).

In muscle composition, similar to the body composition, no significant differences were found in ash content among the groups. Statistically significant but numerically small differences in moisture content were observed among the groups, with the highest and lowest values being recorded in the C5 and C3 groups, respectively. Crude protein content was significantly higher in the C2 and C4 groups compared to that of the C1, C3, and C5 groups (*p* < 0.05). Crude lipid content decreased progressively with increasing dietary carbohydrate levels, with the highest values observed in the C1 and C2 groups and the lowest in the C5 group (*p* < 0.05).

### 3.3. Internal Organ Indices

The viscerosomatic index (VSI), hepatosomatic index (HSI), intestosomatic index (ISI), and intraperitoneal fat ratio (IPF) of *O. bidens* fed diets with varying dietary carbohydrate levels are presented in Table 4. The VSI was significantly lower in the C2 group compared to the C1 and C4 groups (*p* < 0.05), indicating reduced visceral mass relative to body weight. The ISI in the C2 and C3 groups was significantly lower than in the other groups (*p* < 0.05). The HSI increased significantly with rising dietary carbohydrate levels. Notably, fish in the C5 group exhibited a significantly higher HSI than those in the C2 and C3 groups (*p* < 0.05), suggesting enhanced fat deposition in the liver under high carbohydrate intake. Moreover, the IPF indicated a clear increasing trend with dietary carbohydrate levels. Specifically, the IPF in the C1 and C2 groups was significantly lower than that in the C3, C4, and C5 groups (*p* < 0.05).

### 3.4. Serum Biochemistry Parameters

The serum biochemistry parameters of *O. bidens* fed with different diets are shown in Table 5. Serum glucose (GLU), triglyceride (TG), and cholesterol (CHO) levels increased significantly with increasing dietary carbohydrate levels. Notably, the C4 and C5 groups exhibited a particular increase in GLU, TG, and CHO (*p* < 0.05), where GLU, TG, and CHO concentrations were approximately double those detected in the C1 and C2 groups. Serum low-density lipoprotein (LDL) levels were significantly higher in the C1 group compared to the other groups (*p* < 0.05), followed by the C3, C5, C4, and C2 groups in descending order. In contrast, high-density serum lipoprotein (HDL) levels were significantly higher in the C4 and C5 groups compared to the C2, C3, and C1 groups (*p* < 0.05), with the C1 group displaying the lowest HDL concentration. As a result, the LDL/HDL ratio was significantly elevated in the C1 group compared to all other groups (*p* < 0.05).

### 3.5. mRNA Expression Levels of Glucose Metabolism-Related Genes

**Figure 3:**
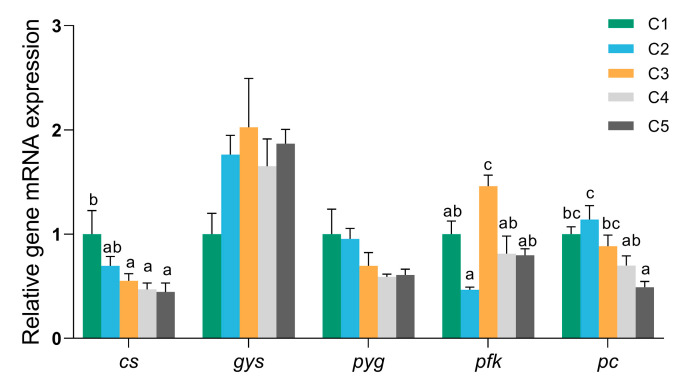
Relative expression of genes related to glucose metabolism in *O. bidens* fed different diets. The expression value was normalized against the expression of the internal control gene β-actin. Fish fed diets containing different levels of α-starch (8%, 14%, 20%, 26%, and 32%) were named as group C1, C2, C3, C4, and C5, respectively. Abbreviations: *cs*: citrate synthase; *gys*: glycogen synthase; *pc*: pyruvate carboxylase; *pfk*: phosphofructokinase; *pyg*: glycogen phosphorylase. Data are presented as mean ± SE of six replicates (*n* = 6). For each gene, the bar with different letters indicates significant differences between groups based on one-way analysis of variance (ANOVA) followed by Duncan’s multiple range test (*p* < 0.05).

The effects of dietary carbohydrate levels on the mRNA expression of key genes involved in glucose metabolism are shown in Figure 3. Citrate synthase (*cs*), a key rate-limiting enzyme in the tricarboxylic acid cycle, exhibited significantly lower mRNA expression levels in fish fed diets with higher carbohydrate levels (C3, C4, and C5 groups) compared to those in the C1 and C2 groups (*p* < 0.05).

Glycogen synthase (*gys*) and glycogen phosphorylase (*pyg*), which catalyze glycogenesis and glycogenolysis, respectively, showed no significant differences in mRNA expression among the groups. However, there was a trend of increased *gys* expression with increased dietary carbohydrate levels, while *pyg* expression displayed the opposite change.

Moreover, dietary carbohydrate levels significantly influenced the mRNA expression of phosphofructokinase (*pfk*) and pyruvate carboxylase (*pc*). The expression of *pfk* was markedly higher in the C3 group compared with the other groups (*p* < 0.05), followed by the C1, C4, and C5 groups; the C2 group exhibited the lowest expression level. *pc* gene expression declined with increasing dietary carbohydrate levels, with the C5 group showing the lowest expression (*p* < 0.05).

### 3.6. mRNA Expression Levels of Lipid Metabolism-Related Genes

**Figure 4:**
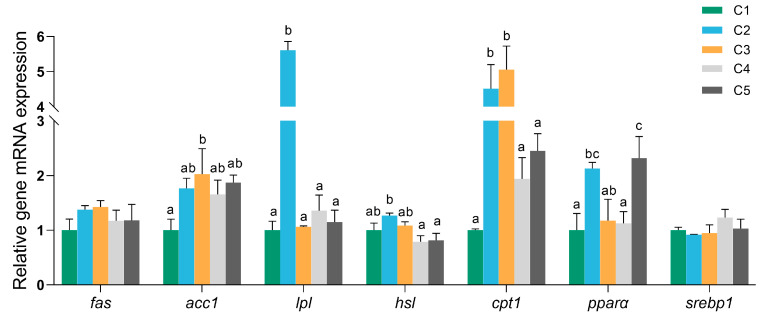
Relative expression of genes related to lipid metabolism in *O. bidens* fed different diets. The expression value was normalized against the expression of the internal control gene β-actin. Fish fed diets containing different levels of α-starch (8%, 14%, 20%, 26%, and 32%) were named as group C1, C2, C3, C4, and C5, respectively. Abbreviations: *fas*: fatty acid synthase; *acc1*: acetyl-coa carboxylase 1; *lpl*: lipoprotein lipase; *hsl*: hormone-sensitive lipase; *cpt1*: carnitine palmitoyltransferase 1; *pparα*: peroxisome proliferator-activated receptor alpha; *srebp1*: sterol regulatory element-binding protein 1. Data are presented as mean ± SE of six replicates (*n* = 6). For each gene, the bar with different letters indicates significant differences between groups based on one-way analysis of variance (ANOVA) followed by Duncan’s multiple range test (*p* < 0.05).

The effects of dietary carbohydrate levels on the hepatic mRNA expression of key genes involved in lipid metabolism are presented in Figure 4. As dietary carbohydrate levels increased, the expression of the acetyl-CoA carboxylase 1 (*acc1*) gene, a key enzyme involved in de novo fatty acid synthesis, was significantly upregulated (*p* < 0.05).

The expression of the lipoprotein lipase (*lpl*) gene, which facilitates triglyceride clearance by hydrolyzing circulating triglycerides into free fatty acids and glycerol, showed dramatically higher expression levels in the C2 group (*p* < 0.05). Similarly, hormone-sensitive lipase (*hsl*), a key enzyme regulating the mobilization of stored triacylglycerols, exhibited the highest expression levels in the C2 group, significantly higher than the levels observed in the C4 and C5 groups (*p* < 0.05). The expression pattern of carnitine palmitoyltransferase 1 (*cpt1*), a rate-limiting enzyme in mitochondrial β-oxidation of fatty acids, exhibited a pattern similar to that of *hsl*, with significantly higher levels in the C2 and C3 groups compared to C1, C4, and C5 (*p* < 0.05).

In addition, the expression of two major transcriptional regulators was differentially affected by carbohydrate levels. Peroxisome proliferator-activated receptor alpha (*pparα*), which promotes fatty acid catabolism, exhibited the highest expression in the C5 group, followed by the C2 group (*p* < 0.05). However, sterol regulatory element-binding protein 1 (*srebp1*), a master regulator of lipogenesis, showed no significant differences among the groups.

## 4. Discussion

*Opsariichthys bidens* has been regarded as an emerging aquaculture fish species in recent years. Although optimal dietary nutrients are essential for fish growth, development, and nutritional quality, the nutritional requirements of *O. bidens* remain poorly understood. Considering the relatively low cost of carbohydrates and their potential role in improving physical properties of feed, the optimal dietary carbohydrate level for *O. bidens* diets should be determined. Previous studies have demonstrated that dietary carbohydrate can contributed to protein sparing in some fish species [18,19,20]. However, the optimal dietary carbohydrate level varies among fish species. Herbivorous and omnivorous fish, such as grass carp (*Ctenopharyngodon idella*) and gibel carp (*Carassius auratus*), can efficiently utilize carbohydrate levels as high as 45% [16,21]. In contrast, carnivorous fish generally tolerate much lower levels; a dietary level of less than 15–25% digestible carbohydrate was found to be appropriate for carnivorous fish. For example, the suitable dietary starch level for juvenile cobia (*Rachycentron canadum* L.) is estimated at 21.1% based on SGR [22], while the optimal dietary carbohydrate level for largemouth bass is 10–19% [14,23]. In small carnivorous fish, Chinese perch (*Siniperca chuatsi*) showed optimal growth performance with dietary starch inclusion levels ranging from 8% to 10% [24]. The dietary digestible carbohydrate levels for gilthead sea bream (*Sparus aurata*) should be less than 20% [25]. In the present study, growth performance of *O. bidens* improved significantly as dietary α-starch levels increased from C1 (8%) to C2 (14%). Both broken-line and polynomial regression analyses based on WGR and SGR consistently indicated an optimal dietary α-starch level of approximately 14–17%, which is considered an appropriate level of digestible carbohydrate for most carnivorous fish. These results suggest that *O. bidens* has a relatively limited capacity to utilize dietary carbohydrate. To ensure isonitrogenous and isolipidic diets, α-starch was replaced with cellulose from 24% (C1) to 0% (C5) when adjusting carbohydrate levels. Although cellulose is poorly digested by fish, it can influence gut motility, satiety, and nutrient absorption. Moreover, previous studies have reported that dietary fiber could modulate the gut environment and microbiota, thereby indirectly affecting digestive efficiency and growth performance [5,26,27,28,29,30]. Thus, while the use of cellulose served to balance nutrient composition, its physiological effects on gut function and digestion should not be ignored.

Body composition analysis revealed distinct tissue-specific responses to varying dietary carbohydrate levels. The whole-body crude protein content exhibited an increase from the C1 to the C3 groups, indicating that moderate carbohydrate levels enhanced overall protein retention. This pattern was further reflected in the muscle tissue, where the C2 group demonstrated a significantly higher crude protein content compared to the C1, C3, and C5 groups. This pattern demonstrates the presence of an optimal carbohydrate range for maximizing protein deposition in muscle in *O. bidens*. The results aligned with previous reports on other carnivorous and omnivorous fish species, including juvenile cobia (*Rachycentron canadum*) [22], hybrid tilapia (*Oreochromis niloticus × O. aureus*) [31], and golden pompano (*Trachinotus ovatus*) [32]. The consistency across different species supports the “protein-sparing” hypothesis that the inclusion of optimal carbohydrate likely provides an available energy source, thereby reducing the catabolism of dietary protein for energy purposes.

The deposition of body lipids was markedly influenced by dietary α-starch in the study. Whole-body crude lipid content exhibited no significant difference among groups C1 to C4, but increased sharply in group C5, whereas muscle crude lipid content displayed the opposite pattern, with the lowest crude lipid content in group C5. The pattern of reduced muscle lipid deposition under high carbohydrate intake has also been reported in other fish species [32,33]. Both the viscerosomatic index (VSI) and intestosomatic index (ISI) were significantly lower in the C2 group than in other groups of *O. bidens*, indicating improved metabolic efficiency and minimized visceral lipid deposition at optimal dietary α-starch levels. Conversely, the hepatosomatic index (HSI) and intraperitoneal fat ratio (IPF) increased significantly with rising dietary carbohydrate levels in *O. bidens*, indicating that when dietary α-starch was supplied in excess, dietary carbohydrate was likely converted into lipids via hepatic lipogenesis that were subsequently deposited as hepatic lipid and intraperitoneal fat in *O. bidens*. An interesting observation in this study was the inverse relationship between dietary carbohydrate level and muscle crude lipid content, despite the concurrent increase in whole-body lipid and HSI. This indicated a redistribution of lipid deposition within the body, where higher dietary carbohydrate levels may promote hepatic lipid synthesis and accumulation while reducing lipid storage in muscle tissue. Similar lipid partitioning patterns have been reported in juvenile tilapia, where a dietary carbohydrate-to-lipid ratio of 1.14 resulted in the highest crude lipid content and the lowest HSI. However, when tilapia were fed high-carbohydrate diets, HSI increased while muscle lipid decreased, suggesting that excessive carbohydrate intake promotes lipid deposition in the liver rather than in muscle tissue [34].

In addition to body composition, plasma biochemical parameters provided further evidence of altered metabolism. Plasma biochemical parameters are strongly influenced by the nutritional status of fish. Many species, particularly carnivorous fish, exhibit chronic postprandial hyperglycemia when fed digestible carbohydrate [8]. In the present study, plasma glucose levels in *O. bidens* were significantly upregulated with increased dietary carbohydrate levels. Moreover, excessive carbohydrate intake (C4: 26.00% α-starch; C5: 32.00% α-starch) elevated plasma triglyceride and cholesterol concentrations, accompanied by a modified lipoprotein profile characterized by decreased LDL and increased HDL levels. Since the balance of lipid metabolism relies on the transport of triglycerides by HDL and LDL to target organs such as the liver [35], the alterations suggested that high dietary carbohydrate intake enhanced lipid production and transport under high-carbohydrate diets. The changes might also explain the observed increases in hepatic lipid accumulation and intraperitoneal fat deposition in *O. bidens* fed high-carbohydrate diets. These findings were consistent with previous reports on blunt snout bream and spotted sea bass (*Lateolabrax maculatus*) [36,37], where high-carbohydrate diets similarly led to modifications in lipid profiles and lipid storage patterns. These studies corroborate our observations and suggest an adaptive response to the increased lipid production and transport, which could contribute to lipid deposition in tissues like the liver and peritoneal fat. These results highlight the need for a more detailed understanding of how high-carbohydrate diets impact lipid metabolism, and their potential implications for metabolic health.

Altering the dietary carbohydrate content of the fish induced noticeable changes in carbohydrate and lipid metabolism, particularly through the regulation of hepatic gene expression [24]. In general, higher dietary carbohydrate intake increased glycolysis capacity, as reflected by the upregulation of the hepatic glycolytic genes glucokinase (*gk*) and phosphofructokinase (*pfk*) in fish including gibel carp, rainbow trout (*Oncorhyncus mykiss*), and gilthead sea bream (*Sparus aurata*) [38,39]. Consistent with these findings, the present study demonstrated that *pfk* expression in *O. bidens* was significantly elevated in the C3 group, but excessive dietary carbohydrate (>26.00% α-starch) did not induce additional upregulation of the *pfk* gene. Hepatic glycogen metabolism was also strongly affected by carbohydrate levels. In this study, high-carbohydrate diets induced a trend of increased *gys* expression with increased dietary carbohydrate levels, while *pyg* expression displayed the opposite change. Similar regulatory patterns have been reported in grass carp that glycogen synthesis-related genes were significantly upregulated under high-carbohydrate feeding, whereas glycogen phosphorylase (*gp*) was inhibited in Chinese long snout catfish (*Leiocassis longirostris*) [40]. Together, these results suggest that high dietary carbohydrate intake consistently promoted glycogen accumulation across different teleost species.

At the mitochondrial level, excessive dietary carbohydrate appears to impair oxidative metabolism. Citrate synthase is a key rate-limiting enzyme in the tricarboxylic acid (TCA) cycle, linking glycolysis-derived acetyl-CoA to mitochondrial ATP production. Previous studies reported that dietary carbohydrate source levels significantly influence *cs* expression and enzyme activity in species such as largemouth bass (*Micropterus salmoides*) and blunt snout bream [36,41]. Specifically, high dietary carbohydrate levels (43%) resulted in a remarkable decrease in activities of CS in blunt snout bream [42]. Similarly, our study found significantly reduced hepatic *cs* mRNA expression in *O. bidens* fed high-carbohydrate diets (>20% α-starch), suggesting a decline in mitochondrial oxidative capacity under excessive carbohydrate. Taken together, these gene expression profiles highlight a coordinated metabolic reprogramming in response to elevated dietary carbohydrates. These transcriptional changes suggest that excessive carbohydrate intake diverted energy metabolism away from mitochondrial oxidation towards enhanced glycogen storage and lipogenesis and ultimately resulted in glycogen and fat accumulation and metabolic disorder in the liver [43,44].

It is well known that glucose can be converted into lipid precursors such as dihydroxyacetone phosphate and acetyl CoA [8]. Many studies have found an association between high-carbohydrate feeding and increased expression and/or activities of some de novo lipogenesis-related enzymes. In juvenile Japanese flounder the mRNA levels of *acaca* and *fasn* and FAS activity were upregulated when fish were fed higher dietary starch levels [45,46]. These results were also reported in juvenile flounder (*Paralichthys olivaceus*) [47], grass carp [48], and Nile tilapia (*Oreochromis niloticus*) [49], which revealed enhanced lipogenic activity in response to elevated carbohydrate intake. Similarly, the expression of the acetyl-CoA carboxylase 1 (*acc1*) gene, a key enzyme involved in de novo fatty acid synthesis, was significantly upregulated with increasing dietary carbohydrate levels in *O. bidens*. The findings were consistent with the elevated TG and CHO levels in the plasma and the lipid accumulation in the liver in our investigation, as previously observed in other studies [50,51]; we assumed that a high-carbohydrate diet might promote lipid synthesis in *O. bidens.* In addition, lipoprotein lipase (*lpl*), hormone-sensitive lipase (*hsl*), and carnitine palmitoyltransferase 1 (*cpt1*), which are the major regulatory genes of lipolysis and mitochondrial β-oxidation, exhibited their highest expression levels in the C2 group. This suggests that moderate carbohydrate intake might facilitate capacity for triglyceride clearance and lipid storage at moderate carbohydrate levels. Similarly, the stimulation of lipolysis and β-oxidation, evidenced by elevated *hsl2* mRNA levels and increased activities of TLP and CPT1, has also been reported in Japanese flounder fed carbohydrate-enriched diets, consistent with previous observations in rainbow trout and gilthead sea bream [45,52,53]. However, further increases in dietary carbohydrate (>26% α-starch) did not induce additional upregulation of these genes in *O. bidens*, indicating a potential suppression of lipolysis and fatty acid oxidation at excessive carbohydrate levels. Similar inhibitory effects of high dietary carbohydrate contents (≥45%) on lipolysis and β-oxidation have also been reported in grass carp [54] and abalone (*Haliotis discus hannai*) [55]. Taken together, these results demonstrate that the dietary carbohydrates promoted fatty acid biosynthesis in *O. bidens*, particularly at higher inclusion levels. Nevertheless, excessive carbohydrate intake might contribute to lipid deposition by upregulating the expressions of lipid synthesis-related genes and reducing the expressions of lipolysis- and β-oxidation-related genes.

## 5. Conclusions

In conclusion, *O. bidens* exhibited a limited capacity to utilize dietary carbohydrate, with the optimal α-starch level for growth estimated at 14–17%. Optimal carbohydrate intake promoted glycolysis and glycogen synthesis and supported balanced glucose and lipid metabolism. In contrast, excessive carbohydrate intake suppressed lipolysis and fatty acid oxidation while stimulating hepatic lipogenesis and glycogen accumulation, ultimately leading to lipid deposition and metabolic disorders. These findings provide a nutritional basis for optimizing carbohydrate inclusion in *O. bidens* diets and highlight the importance of maintaining appropriate carbohydrate levels to avoid metabolic disorders.

## Figures and Tables

**Table 1:** Ingredients and feed formulation of experimental diets.

Item	C1	C2	C3	C4	C5
**Ingredients %**					
Fish meal	26.00	26.00	26.00	26.00	26.00
Casein ^(1)^	28.50	28.50	28.50	28.50	28.50
Fish oil	2.75	2.75	2.75	2.75	2.75
Soybean oil	2.75	2.75	2.75	2.75	2.75
α-starch ^(2)^	8.00	14.00	20.00	26.00	32.00
Vitamin and mineral mix ^(3)^	4.00	4.00	4.00	4.00	4.00
Cellulose	24.00	18.00	12.00	6.00	0.00
Ca(H_2_PO_4_)_2_	1.50	1.50	1.50	1.50	1.50
Choline chloride	0.50	0.50	0.50	0.50	0.50
Sodium carboxymethylcellulose	2.00	2.00	2.00	2.00	2.00
**Compositions**					
Dry matter (DM) (%)	95.22	94.35	94.86	95.61	94.12
Crude protein (% DM)	42.05	42.05	42.05	42.05	42.05
Crude lipid (% DM)	7.85	7.85	7.85	7.85	7.85
Ash (% DM)	14.22	15.47	13.64	14.65	14.33
Nitrogen-free extract (% DM)	10.68	15.55	23.81	29.17	35.77
Gross energy (Mcal/kg)	274.69	298.69	322.69	346.69	370.69

^(1)^ Crude protein and lipid content of casein was 84.4% and 0.6%, respectively. ^(2)^ Corn starch was the sole starch source. Crude protein and lipid content of α-starch was 0.3% and 0.2%, respectively. ^(3)^ Vitamin premix: VA, 1000 IU; VD3, 3000 IU; VE, 150 IU; VK3, 12.17 mg; VB1, 20 mg; VB2, 20 mg; VB3, 100 mg; VB6, 22 mg; VB12, 0.15 mg; VC, 300 mg; biotin, 0.6 mg; inositol, 400 mg; folic acid, 8 mg. Mineral premix: I, 1.5 mg; Mn, 11.45 mg; Co, 0.6 mg; Cu, 3 mg; Zn, 89 mg; Se, 0.24 mg; Mg, 180 mg; Fe, 63 mg.

**Table 2:** The primers for RT-qPCR.

Gene	Primer Sequences (5′ to 3′)	Tm (°C)
*fas*	F: GGCATCTGGAGGCAGTTTG	55.0
	R: GTGAATGTCCTGACCCGTG	
*acc1*	F: TGTTGTTGTTTGTCCCTCCTG	55.0
	R: TACTGTCGCTTCCCCCG	
*lpl*	F: GGATAATAAGGAAGGTTTGGGAA	55.0
	R: AGCAGACAGTGGACTACAGTGACA	
*hsl*	F: TTGTTCACAGTCGTGTCGTCT	55.0
	R: GATTTCATTGGCTCAAGGTT	
*cpt1*	F: GCAGAAACCGCACGAATC	55.0
	R: GTGGCAGCGAACAACAGTC	
*pparα*	F: GTGACCTCGCACTGTTTGT	55.0
	R: CTGGGTGGTTGCTCTTTAG	
*srebp1*	F: GAGTATTCCCCGTCCCC	55.0
	R: CCTGTCTCTCGCCAAGC	
*cs*	F: TCTCACTGTTCAGAGCCGT	55.0
	R: ATCCGTTTCCGTGGTTAC	
*gys*	F: GGCGGGAGCGGCACAG	55.0
	R: ACGACAGGGAGGCGAACGA	
*pc*	F: TTCACAGAGTCAGCAGTCAGTC	55.0
	R: GGCGATAAATACGGCAAC	
*pfk*	F: TGTGACCGAATCAAGCAATC	55.0
	R: GCCCCAGCCGCTAAACC	
*pyg*	F: GGAGGGGAAGGAGCCG	55.0
	R: GGAGATTGAAGTCGTTGGGA	
*β-actin*	F: CAAACCCCCCCAAACCTA	55.0
	R: GAGCATCATCTCCAGCGAAT	

*fas*: fatty acid synthase; *acc1*: acetyl-coa carboxylase 1; *lpl*: lipoprotein lipase; *hsl*: hormone-sensitive lipase; *cpt1*: carnitine palmitoyltransferase 1; *pparα*: peroxisome proliferator-activated receptor alpha; *srebp1*: sterol regulatory element-binding protein 1; *cs*: citrate synthase; *gys*: glycogen synthase; *pc*: pyruvate carboxylase; *pfk*: phosphofructokinase; *pyg*: glycogen phosphorylase; *β-actin*: beta-actin.

**Table 3:** Whole body and muscle composition of fish fed diets with different levels of α-starch.

	C1	C2	C3	C4	C5	*p*-Value
Body composition (%)						
Moisture	73.42 ± 1.26	72.75 ± 0.56	72.55 ± 0.35	72.73 ± 0.65	71.76 ± 0.94	0.772
Crude Protein	15.90 ± 0.06 ^ab^	16.30 ± 0.11 ^ab^	16.67 ± 0.05 ^b^	15.61 ± 0.20 ^a^	18.17 ± 0.60 ^c^	1.2 × 10^−5^
Crude Lipid	8.30 ± 0.16 ^a^	12.85 ± 0.01 ^b^	10.62 ± 0.01 ^ab^	12.98 ± 0.01 ^b^	32.5 ± 0.14 ^c^	1.782 × 10^−10^
Ash	2.38 ± 0.04	2.43 ± 0.12	2.36 ± 0.03	2.41 ± 0.16	2.40 ± 0.04	0.528
Muscle composition (%)						
Moisture	77.42 ± 0.25 ^ab^	78.14 ± 0.14 ^ab^	77.21 ± 0.64 ^a^	77.44 ± 0.18 ^ab^	78.38 ± 0.22 ^b^	0.048
Crude Protein	17.80 ± 0.01 ^a^	18.20 ± 0.20 ^b^	17.68 ± 0.15 ^a^	18.18 ± 0.06 ^b^	17.73 ± 0.02 ^a^	0.011
Crude Lipid	7.23 ± 0.2 ^c^	6.64 ± 0.2 ^bc^	4.36 ± 0.2 ^ab^	4.63 ± 0.2 ^ab^	3.74 ± 0.2 ^a^	0.037
Ash	1.35 ± 0.24	1.36 ± 0.26	1.41 ± 0.23	1.34 ± 0.04	1.38 ± 0.05	0.414

Data are presented as mean ± SE of six replicates (*n* = 6). The same row with different letters indicates significant differences between groups based on one-way analysis of variance (ANOVA) followed by the Duncan’s multiple range test (*p* < 0.05).

**Table 4:** Internal organ indices of fish fed diets with different levels of α-starch.

	C1	C2	C3	C4	C5	*p*-Value
VSI	9.27 ± 0.32 ^b^	7.82 ± 0.29 ^a^	8.87 ± 0.30 ^ab^	9.71 ± 0.70 ^b^	8.61 ± 0.41 ^ab^	0.049
ISI	2.71 ± 0.12 ^b^	1.76 ± 0.08 ^a^	2.00 ± 0.11 ^a^	2.49 ± 0.14 ^b^	2.60 ± 0.13 ^b^	7.0 × 10^−5^
HSI	0.82 ± 0.10 ^a^	0.70 ± 0.01 ^a^	0.58 ± 0.12 ^a^	0.77 ± 0.13 ^ab^	1.18 ± 0.23 ^b^	0.031
IPF	2.48 ± 0.45 ^a^	2.30 ± 0.27 ^a^	3.35 ± 0.35 ^b^	2.89 ± 0.83 ^ab^	3.33 ± 0.46 ^b^	0.043

Data are presented as mean ± SE of six replicates (*n* = 6). The same row with different letters indicates significant differences between groups based on one-way analysis of variance (ANOVA) followed by the Duncan’s multiple range test (*p* < 0.05).

**Table 5:** Serum biochemistry parameters of fish fed diets with different levels of α-starch.

	C1	C2	C3	C4	C5	*p*-Value
GLU (mmol/L)	2.12 ± 0.21 ^a^	2.52 ± 0.34 ^a^	3.78 ± 0.28 ^b^	6.10 ± 0.11 ^c^	7.49 ± 0.09 ^d^	4.21 × 10^−8^
TG (mmol/L)	0.97 ± 0.15 ^a^	0.97 ± 0.05 ^a^	1.15 ± 0.06 ^ab^	1.28 ± 0.04 ^bc^	1.46 ± 0.09 ^c^	0.011
CHO (mmol/L)	3.09 ± 0.41 ^a^	3.57 ± 0.66 ^a^	3.62 ± 0.12 ^a^	8.42 ± 0.43 ^b^	8.62 ± 0.14 ^b^	1.07 × 10^−5^
LDL (mmol/L)	1.63 ± 0.38 ^b^	0.36 ± 0.08 ^a^	0.95 ± 0.14 ^a^	0.44 ± 0.10 ^a^	0.65 ± 0.15 ^a^	0.007
HDL (mmol/L)	1.88 ± 0.58 ^a^	3.59 ± 0.38 ^ab^	2.82 ± 0.16 ^a^	5.43 ± 0.36 ^c^	5.03 ± 0.88 ^bc^	0.004
LDL/HDL	0.99 ± 0.28 ^b^	0.10 ± 0.01 ^a^	0.35 ± 0.07 ^a^	0.08 ± 0.03 ^a^	0.15 ± 0.06 ^a^	0.003

Data are presented as mean ± SE of six replicates (*n* = 6). The same row with different letters indicates significant differences between groups based on one-way analysis of variance (ANOVA) followed by the Duncan’s multiple range test (*p* < 0.05).

## Data Availability

The data presented in this study are available on request from the corresponding author. The data are not publicly available because they form part of an ongoing research project and will be used for subsequent analyses and future publications.
